# Determinants of high-risk fertility behavior among women of reproductive age in Kenya: a multilevel analysis based on 2022 Kenyan demographic and health survey

**DOI:** 10.1186/s12889-023-17459-w

**Published:** 2023-12-15

**Authors:** Beminate Lemma Seifu, Tsion Mulat Tebeje, Yordanos Sisay Asgedom, Zufan Alamrie Asmare, Hiwot Altaye Asebe, Bizunesh Fantahun Kase, Abdu Hailu Shibeshi, Kebede Gemeda Sabo, Bezawit Melak Fente, Kusse Urmale Mare

**Affiliations:** 1 Department of Public Health, College of Medicine and Health Sciences, Samara University, Semera, Ethiopia; 2 School of Public Health, College of health sciences and Medicine, Dilla University, Dilla, Ethiopia; 3 Department of Epidemiology and Biostatics, College of Health Sciences and Medicine, Wolaita Sodo University, Soddo, Ethiopia; 4 Department of Ophthalmology, School of Medicine and Health Science, Debre Tabor University, Debre Tabor, Ethiopia; 5 Department of Public Health, Collage of Medicine and Health Sciences, Samara University, Semera, Ethiopia; 6 Department of Statistics, College of Natural and Computational Science, Samara University, Semera, Ethiopia; 7 Department of Nursing, College of Medicine and Health Sciences, Samara University, Semera, Ethiopia; 8 Department of Clinical Midwifery, School of Midwifery, College of Medicine & Health Sciences, University of Gondar, Gondar, Ethiopia

**Keywords:** High-risk fertility behavior, Kenya, Multi-level analysis, Demographic and health survey

## Abstract

**Background:**

Women’s high-risk fertility behavior (HRFB), which is characterized by narrow birth intervals, high birth order, and younger maternal age at birth, have been scientifically reported to have detrimental effects on the mother and child’s health. To date, there has been limited research into the underlying factors contributing to high-risk fertility behavior in Kenya. Thus, the aim of this study is to identify the factors associated with high-risk fertility behavior among women of reproductive age in Kenya.

**Method:**

The 2022 Kenyan Demography and Health Survey data was used for the current study. This study included 15,483 women of reproductive age. To account for the clustering effects of DHS data and the binary nature of the outcome variable, a multilevel binary logistic regression model was applied. An adjusted odds ratio with a 95% confidence interval was reported to declare the statistical significance. In addition, the model that had the lowest deviance was the one that best fit the data.

**Results:**

The overall prevalence of HRFB among Kenyan women were 70.86% (95%CI = 69.96, 71.40). Women with primary, secondary, and higher educational levels, Protestant and Muslim religion followers, women whose husbands/partners had secondary and higher educational levels, a high household wealth index, ever had a terminated pregnancy, and rural residence, all of these factors were found to be strongly associated with high-risk fertility behavior.

**Conclusion:**

As per the findings of our study, in Kenya a significant proportion of women has experienced HRFB. This is a matter of concern as it poses a significant challenge to the healthcare system. The high prevalence of HRFB indicates that there is an urgent need to take appropriate measures in order to mitigate its impact. The situation calls for a comprehensive and coordinated approach involving all stakeholders to address this issue effectively. It would benefit policymakers to create programs that consider factors like education, wealth, and residence that make women more susceptible to HRFB. Targeting women living in high HRFB-prevalence areas could help address the root causes of the issue. This approach can alleviate negative impacts and ensure effective and sustainable solutions.

## Introduction

Sub-Saharan Africa (SSA) and Southern Asia accounted for approximately 87% (253,000) of the expected global maternal deaths in 2020. Around 70% (202,000) of maternal deaths were attributed to SSA alone [1]. One of the objectives of Sustainable Development Goals (SDG) 3 is to reduce the global maternal mortality rate (MMR) to less than 70 per 100,000 births. Additionally, no country should have a maternal mortality rate that is more than twice the global average [2]. In 2020, the worldwide maternal mortality ratio (MMR) was 223 for every 100,000 live births. To achieve a global MMR of less than 70 per 100,000 live births by 2030, a yearly reduction rate of 11.6% is necessary. This rate has rarely been achieved at the national level [3].

According to 2022’s report the world population is rapidly expanding with a total fertility rate of 2.5 and 3.4 per woman globally and in Kenya, respectively [4]. Kenya has a high maternal mortality ratio of 355 deaths per 100,000 live births, which means that there are nearly 5000 women and girls dying annually [5]. In Kenya and other regions of Sub-Saharan Africa, high-risk fertility behaviors remain a contributing factor for poor maternal health outcomes, including maternal mortality risks [6].

Women’s high-risk fertility behavior (HRFB), which is characterized by “narrow birth intervals, high birth order, and younger maternal age at birth”, have been scientifically reported to have detrimental effects on the mother’s and child’s health [7, 8]. Infant and child survival is partly influenced by their mothers’ demographic and biological factors [9]. Statistically, children born to mothers who are either too young (below 18) or too old (above 34), children born within a short birth interval (less than 24 months after the previous birth), and children born to mothers with a high parity (more than three children) are at a higher risk of mortality [10–12].

Getting pregnant before the age of 18 can lead to various complications, premature birth and low birth weight are both risks throughout pregnancy and childbirth. Additionally, teenage mothers have a higher likelihood of developing anemia [13, 14]. Pregnancy at an older age can lead to higher risks of complications for both the mother and the baby, such as stillbirth, a smaller-sized baby for gestational age, preeclampsia, and maternal death [15]. Studies demonstrated that a short birth interval can raise the likelihood of adverse perinatal health outcomes and congenital abnormalities [16, 17].

While there is evidence to support the importance of considering various exposures to high-risk fertility behaviors as a top priority for maternal and child health, there have been very few studies conducted in Kenya that specifically examine the factors related to HRFB among women of reproductive age. To develop effective prevention programs for the region, it is important to have a clear understanding of the determinants and potential risk factors for maternal high-risk fertility behavior among women in Kenya. However, there is a paucity of literature evaluating the risk factors for HRFB in Kenya. With regard to these considerations, the aim of this study was to identify the risk factors for HRFB among RAW. It will be essential to identify these determinants to develop evidence-based programs in Kenya, specifically targeting the significant risk factors.

## Method

### Study design, data source and setting

Kenya Demographic and Health Survey (KDHS) was the seventh survey undertaken in Kenya, preceding similar surveys. The 2022 Kenya Demographic and Health Survey (KDHS) utilized a two-stage stratified sampling design. In the first stage, 1,692 clusters were selected from the Kenya Household Health Survey Framework (KHHSF) using the Equal Probability Selection Method (EPSEM). The survey includes multiple datasets for men, women, children, births, and households. We used the Individual Record dataset (IR file) for this study. Reproductive-age women from Kenya’s population were selected as the source, while those from designated EAs were chosen as the study population. A total weighted sample of 15,483 reproductive-age women was considered for the final analysis. Detailed information about DHS methodology can be found from the official database https://dhsprogram.com/Methodology/index.cfm.

### Study variables and measurements

#### Dependent variable

Three factors were used as indicators of high-risk fertility behavior; mothers age at their first birth, birth interval, and birth order. These indicators were dichotomized; we gave 1 if any single risk factors were present and 0 otherwise. We used the KDHS definition of “high-risk fertility behaviors” from 2022 report. The presence of any of the four characteristics listed below was classified as a single high-risk fertility behavior:

1. Mother’s age less than 18 years at the time of childbirth.
2. Mother’s age greater than 34 years at the time of childbirth.
3. Short birth interval (latest child born less than 24 months after the previous birth).
4. The index child’s birth order three or higher.

Two or more of the aforementioned conditions makes up multiple HRFB categories. HRFB is defined as the presence of any listed condition.

#### Independent variables

Maternal education status, husband’s/partner’s educational status, maternal employment status, religion, residence, media exposure, maternal occupation, sex of household head, household wealth status, ever had a terminated pregnancy, contraceptive utilization and women’s decision-making autonomy were the independent variables extracted for this study based on reviewed literatures.

### Data management and analysis

To ensure accurate statistical analysis, we applied weightings to the data based on sampling weight, primary sampling unit, and strata. This was done to restore the survey’s representativeness and account for the sampling design when computing standard errors. The aim was to obtain reliable statistical estimates. STATA version 17 statistical software was used for data management, descriptive statistics, and multilevel binary logistic regression analysis.

To account for the clustering effects of DHS data a multilevel binary logistic regression model was applied to determine the effects of each independent variable on women’s HRFB. Bivariable multilevel binary logistic regression analysis was done to identify variables eligible for the multivariable analysis. Variables with a *p*-value less than 0.20 in this analysis and those found important in the literature were considered as candidates for multivariable multilevel binary logistic regression analysis.

### Model building

High risk fertility behavior is a binary outcome Y_*ij*_ from the sample of individuals *j* = 1,…… from a set of cluster/EA *i*, a set of individual exposures {z_*iju*_; u = 1,2, ….} and a set of cluster-level exposures {X_*ir*_;*r* = 1,2…}.

$${Y}_{ij} \sim {Bernoulli (P}_{ij}$$)

Null model: $$logit\left({P}_{ij}\right)= \mu +{u}_{i}$$

Model 1: $$logit\left({P}_{ij}\right)= \mu + \beta {X}_{ij} +{u}_{i}$$

Mode1 2: $$logit\left({P}_{ij}\right)= \mu + \gamma {Z}_{i}+{u}_{i}$$

Model 3: $$logit\left({P}_{ij}\right)= \mu + \beta {X}_{ij}+ \gamma {Z}_{i}+{u}_{i}$$

To evaluate heterogeneity among clusters, we calculated the Likelihood Ratio (LR) test, Intra-class Correlation Coefficient (ICC), and Median Odds Ratio (MOR). The ICC measures the degree of heterogeneity between clusters by assessing the proportion of individual variation in HRFB among RAW.

ICC= ϭ^2^/ (ϭ^2^+π^2^/3) [18].

The MOR quantifies the variation or heterogeneity in HRFB between clusters in terms of the odds ratio scale and is defined as the median value of the odds ratio between the cluster with a high likelihood of HRFB and the cluster at lower risk when randomly picking out individuals from two clusters (EAs).

MOR = exp $$\sqrt{ \left(2*\partial 2*0.6745\right) }$$$$\sim$$MOR = exp (0.95*$$\partial$$) [19].

$\partial$^2^ indicates that cluster variance.

Variables with a *p*-value < 0.2 in the bi- variable multilevel binary logistic regression analysis were considered for the multivariable analysis. Four models were constructed for the multivariable multilevel binary logistic regression. The first model was a null model without explanatory variables to determine the extent of cluster variation in HRFB. The second model was fitted with individual-level variables, the third with community-level variables, and the fourth with both individual and community-level variables at the same time. Deviance was used to verify model fitness and a model with the lowest deviance was considered the best-fit model. Finally, the Adjusted Odds Ratio (AOR) with its 95% confidence interval (CI) was reported, and variables with a *p*-value < 0.05 in the multivariable analysis were considered as statistically significant predictors.

### Ethical consideration

This study did not require ethical approval or participant consent because it was a secondary data analysis of publicly available survey data from the MEASURE DHS program. We have obtained permission to download and use the data from http://www.dhsprogram.com for this study. There are no names or addresses of individuals or households recorded in the datasets.

## Result

### Descriptive characteristics of the participants

A total of 15,483 women who had given birth within 5 years preceding the survey included in this study. Most women (62.66%) lived in rural areas, and one-thirds (31.54%) had attained primary education. About 6,547 (42.28%) women were found in the age groups of 35–49 years followed by the age groups of 25–34 years (41.78%). The vast majority of mothers (88.72%) had media exposure and two-thirds (61.79%) reported contraceptive utilization (Table 1).

**Table 1:** Distribution of the study population by socio-demographic and reproductive related characteristics (n = 15,483)

Variables	Weighted frequency (%)	High-risk fertility behaviour	
		No (%)	Yes (%)
**Proportion of HRFB 95%CI (70.86% (95%CI = 69.96, 71.40)**			
**Individual level variables**			
**Maternal educational status**			
No formal education	1,295 (8.36)	116 (8.97)	1,179 (91.03)
Primary	6,448 (41.65)	980 (15.20)	5,467 (84.80)
Secondary	4,883 (31.54)	1,999 (40.95)	2,883 (59.05)
Higher	2,857 (18.45)	1,443 (50.49)	1,414 (49.51)
**Husband educational status**			
No formal education	1,172 (7.57)	144 (12.27)	1,028 (87.73)
Primary	5,845 (37.75)	1,024 (17.52)	4,820 (82.48)
Secondary	1,205 (7.78)	1,703 (34.54)	3,228 (65.46)
Higher	7,260 (46.90)	1,667 (47.16)	1,867 (52.84)
**Maternal age in years**			
15–24	2,467 (15.94)	1,186 (48.06)	1,282 (51.94)
25–34	6,469 (41.78)	2,489 (38.48)	3,980 (61.52)
35–49	6,547 (42.28)	863 (13.19)	5,682 (86.81)
**Maternal employment status**			
Not employed	6,339 (40.94)	1,913 (30.17)	4,426 (69.83)
Employed	9,144 (59.06)	2,625 (28.71)	6,518 (71.29)
**Religion**			
Catholic	2,701 (17.45)	905 (33.52)	1,796 (66.48)
Protestant	5,691 (36.75)	1,708 (30.02)	3,982 (69.98)
Evangelical churches	3,754 (24.25)	1,092 (29.11)	2,661 (70.89)
African instituted churches	1,376 (8.89)	367 (26.69)	1,009 (73.31)
Muslim	696 (4.49)	227 (17.99)	1,037 (82.01)
Other	1,264 (8.17)	236 (33.99)	459 (66.01)
**Sex of household head**			
Male	12,116 (78.26)	3,606 (29.76)	8,511 (70.24)
Female	3,366 (21.74)	932 (27.70)	2,433 (72.30)
**Household wealth status**			
Poor	5,489 (35.46)	946 (17.24)	4,543 (82.76)
Middle	2,920 (18.86)	737 (25.25)	2,183 (74.75)
Rich	7,073 (45.68)	2,854 (40.35)	4,218 (59.65)
**Media exposure**			
No	1,747 (11.28)	287 (16.46)	1,459 (83.54)
Yes	13,736 (88.72)	4,251 (30.95)	9,485 (69.05)
**Women’s health care decision-making autonomy**			
Respondent alone	6,397 (41.32)	1,792 (28.02)	4,605 (71.98)
Jointly	6,852 (44.26)	2,185 (31.88)	4,667 (68.12)
Husband, parent or someone else	2,234 (14.42)	561 (25.12)	1,672 (74.88)
**Current contraceptive use**			
No	5,916 (38.21)	1,613 (27.27)	4,303 (72.73)
Yes	9,567 (61.79)	2,925 (30.58)	6,641 (69.42)
**Ever had a terminated pregnancy**			
No	12,658 (81.75)	3,743 (29.57)	8,914 (70.43)
Yes	2,824 (18.25)	795 (28.14)	2,030 (71.86)
**Community level variables**			
**Residence**			
Urban	5,782 (37.34)	2,296 (39.71)	3,485 (60.29)
Rural	9,701 (62.66)	2,242 (23.11)	7,459 (76.89)

### The prevalence of high-risk fertility behavior and its distribution across the independent variables

The overall prevalence of high-risk fertility behavior among Kenyan reproductive age women were 70.86% (95%CI = 69.96, 71.40). The proportion of HRFB among women who do not attained a formal education were 91.03% and 49.51% among women who had higher educational status. Furthermore, the prevalence of HRFB among women from poor household and no media exposure were 82.76% and 83.54%, respectively (Table 1).

**Fig. 1:**
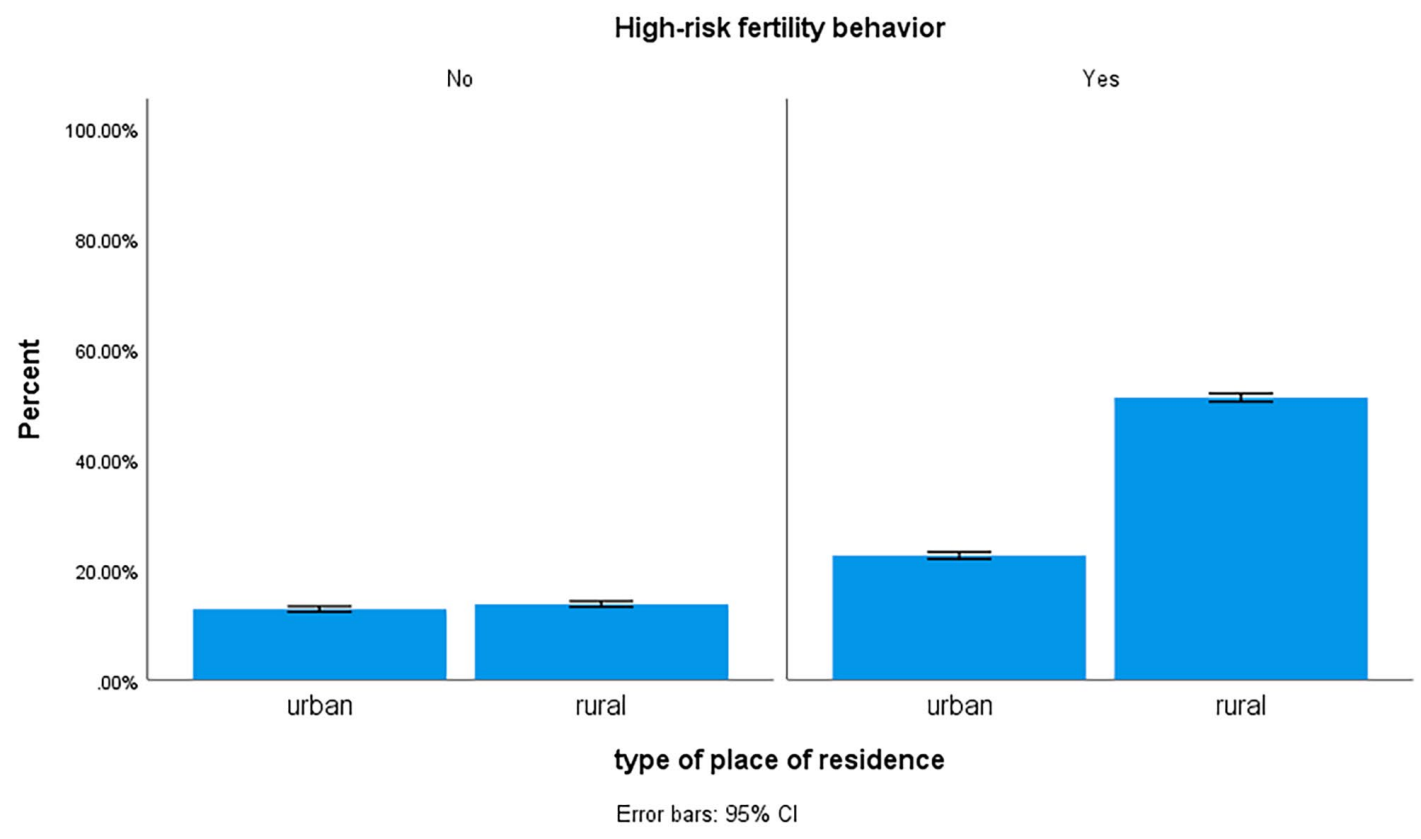
Distribution of high-risk fertility behaviour across residence (n = 15,483)

### Statistical analysis and model comparison

Table 2 shows that the ICC in the null model was 13.6%, indicating that 13.6% of the overall variability for HRFB was related to variations between clusters/EA. Furthermore, the MOR for HRFB in the null model was 1.32, indicating that there was variability between clusters. If we randomly selected an individual from two different clusters, those in the cluster with a higher risk of HRFB had 1.32 times the odds of having HRFB compared to those in the cluster with a lower risk of HRFB. The best-fitted model was chosen based on the lowest deviance value (16,598.74) (Table 2).

### Factors associated with high-risk fertility behavior among reproductive age women in Kenya

In the multivariable mixed effect binary logistic regression model, woman’s educational status, husband’s educational status, religion, wealth index, history of terminated pregnancy and place of residence were found to be statistically significant predictors of HRFB among Kenyan reproductive age women. The odds of having HRFB among women who had primary, secondary and higher educational status were decreased by 47% (AOR = 0.54, 95%CI: 0.44, 0.66), 83% (AOR = 0.17, 95%CI: 0.14, 0.21), and 87% (AOR = 0.13, 95%CI: 0.10, 0.16) respectively, compared to women who do not attend formal education. Protestant and Muslim religion followers had a 1.20 (AOR = 1.20, 95%CI: 1.07, 1.34) and 1.19 (AOR = 1.19, 95%CI: 1.12, 1.41) times higher odds of experiencing HRFB compared to Catholic religion followers. Women whose husband/ partner had a secondary and higher educational status had an odds of HRFB decreased by 21% (AOR = 0.79, 95%CI: 0.65, 0.95) and 26% (AOR = 0.74, 95%CI: 0.60, 0.91) respectively, compared to women whose husband/ partner do not have a formal education. In contrast to women who resides in rich households, women whose household wealth index is middle and poor had 1.21 times (AOR = 1.21, 95%CI: 1.07, 1.37) and 1.22 times (AOR = 1.22, 95%CI: 1.07, 1.39) higher odds of HRFB, respectively. Women who ever had a terminated pregnancy had 1.11 times (AOR = 1.11, 95%CI: 1.01, 1.23) higher risk of HRFB, compared to women who do not had previously terminated pregnancy. The chance of HRFB among rural resident women were1.26 times (AOR = 1.26, 95%CI: 1.13, 1.41) higher than women who resides in urban (Table 2).

**Table 2:** Multilevel analysis of factors associated with high-risk fertility behavior among reproductive age women in Kenya, 2022

Variables	Null model	Model I AOR (95%CI)	Model II AOR (95%CI)	Model III AOR (95%CI)
**Maternal educational status**				
No formal education		1		1
Primary		0.54 (0.44, 0.65)		0.54 (0.44, 0.66)*
Secondary		0.17 (0.14, 0.21)		0.17 (0.14, 0.21)*
Higher		0.13 (0.10, 0.16)		0.13 (0.10, 0.16)*
**Religion**				
Catholic		1		1
Protestant		1.21 (1.08, 1.35)		1.20 (1.07, 1.34)*
Evangelical churches		1.08 (0.96, 1.22)		1.08 (0.96, 1.23)
African instituted churches		1.13 (0.96, 1.34)		1.14 (0.96, 1.34)
Muslim		1.15 (0.98, 1.35)		1.19 (1.12, 1.41)*
Other		0.69 (0.57, 0.86)		0.71 (0.58, 0.87)*
**Husband educational status**				
No formal education		1		1
Primary		1.12 (0.93, 1.35)		1.12 (0.93, 1.35)
Secondary		0.86 (0.68, 1.07)		0.79 (0.65, 0.95)*
Higher		0.76 (0.62, 0.92)		0.74 (0.60, 0.91)*
**Household wealth status**				
Rich		1		1
Middle		1.36 (1.22, 1.52)		1.21 (1.07, 1.37)*
Poor		1.41 (1.26, 1.58)		1.22 (1.07, 1.39)*
**Media exposure**				
No		1		1
Yes		1.08 (0.94, 1.25)		1.07 (0.93, 1.24)
**Current contraceptive use**				
No		1		1
Yes		1.1 (0.98, 1.16)		1.07 (0.98, 1.16)
**Ever had a terminated pregnancy**				
No		1		1
Yes		1.11 (1.00, 1.23)		1.11 (1.01, 1.23)*
**Residence**				
Urban			1	1
Rural			2.26 (2.05, 2.49)	1.26 (1.13, 1.41)*
**Model comparison and random effect results**				
Log-likelihood	-9303.711	-8308.14	-9173.59	-8299.37
Deviance	18,607.42	16,616.28	18,347.18	16,598.74
AIC	18611.42	16652.28	18353.18	16636.74
BIC	18626.84	16791.03	18376.3	16783.2
ICC (95%CI)	13.61(11.80, 15.63)			
MOR (95%CI)	1.32 (1.20, 1.42)			

*p-value < 0.05

*ICC: Intraclass correlation coefficient

*MOR: Median odds ratio

## Discussion

According to the current study, 70.86% (95%CI = 69.96, 71.40) of Kenyan women had a high-risk fertility behavior. Among all women, the most common single risky behavior is having a high birth order, reported by 57.15% of them. On the other hand, the least common single risky behavior, which only affects 5.00% of women, is giving birth for the first time at the age of 34 or older. The high prevalence indicates that HRFB is a widespread issue in Kenya, which could pose a threat to the health of women in the country.

Compared to women and husbands who do not had a formal education, women who had formal education had a lower likelihood of HRFB. This is supported by previous studies [20–22]. Education plays a significant role in promoting autonomy, especially for women. It grants them the ability to delay marriage, negotiate family planning with their partners, and engage with healthcare providers effectively. As a result, education empowers women to achieve their preferences autonomously [23–25].

Our study corroborates the findings from previous studies done in Ethiopia [22], Bangladesh [26] and India [27], which have reported Religious beliefs have been shown to significantly affect HRFB among women, particularly among those who are identifed as Muslim or Protestant. In various regions, these beliefs can shape attitudes towards marriage, reproduction, and contraception, leading to greater engagement in HRFB [28]. Studies indicate that Islamic religious teachings often criticize the use of contraception, leading to shorter intervals between births and higher birth rates [29].

Consistent with previous studies [30, 31], women who belong to households with a lower wealth index have higher chances of experiencing HRFB compared to women from rich households. This finding indicates that women who come from socioeconomically disadvantaged backgrounds may encounter challenges in obtaining necessary health information, possess lower levels of awareness regarding family planning, and experience limited autonomy over the timing and number of children they have. These challenges can eventually impair their ability to live a healthy life, including the appropriate intervals between births, the number of children, and the age of the first pregnancy.

The current study has shown that women with a history of abortion (terminated pregnancy) are more likely to engage in high-risk fertility behaviors compared to those who have not undergone the procedure. This conclusion is in line with previous studies conducted in SSA [32, 33]. Unwanted pregnancies, shorter birth periods, and pregnancies at a young age were often linked to abortion. Additionally, the absence of contraception use had a negative impact on high-risk fertility. Women who struggled to access healthcare were more likely to engage in risky fertility behaviors, which is consistent with previous studies [34].

Compared to women who reside in urban, women who lives in rural areas were found to have increased probability of HRFB. This finding is consistent with studies conducted in East Africa [35], Bangladesh [26], and Nepal [36]. This is most likely owing to the fact that women in rural areas may lag behind in terms of utilizing reproductive health care such as ANC, having low rates of family planning acceptance due to religious beliefs and community attitudes, and having low literacy levels.

### Strength and limitation of the study

This research is one of the few that examine the prevalence and determinants high-risk fertility behavior using the latest DHS data with a large sample size, that were representative of the national population. Furthermore, this study used a weighted dataset with powerful statistical analytic techniques, which attribute the correlated nature of the DHS data and provides us with reliable estimates and standard errors. This study, however, is not without limitations. Because the DHSs are cross-sectional, we cannot prove the causal relationship between the independent variables and the outcome. Furthermore, because the data gathered through interviews, there is a risk of recall bias, and this study does not distinguish between spontaneous and induced abortion.

## Conclusion

Based on the research conducted, it has been determined that Kenya has a high prevalence of high-risk fertility practices. Lower HRFB was associated with multiple significant protective factors, including maternal education, partners’ higher education, and a high household income index. On the contrary, being Muslim and a protestant religion follower, having previously terminated pregnancy and being a rural dweller found to raise a woman’s likelihood of experiancing HRFB. In order to reduce high-risk reproductive behavior and its consequences, It is imperative that policymakers and stakeholders give due consideration to the risk factors associated with high-risk fertility behaviors among women, especially those residing in areas with a high prevalence of such behaviors.

## Data Availability

All result-based data are in the manuscript. In addition, the dataset can be accessed from the measure DHS Program through https://www.dhsprogram.com.

## Abbreviations

AIC: Akaike Information Criteria
AOR: Adjusted Odds Ratio
BIC: Bayesian Information Criteria
CI: Confidence Interval
DHS: Demographic health survey
EAs: Enumeration areas
LMICs: Lower and Middle-income Countries
LLR: Log likelihood ratio
LR: Likelihood ratio
RAW: Reproductive Age Women
SSA: Sub-Saharan Africa
WHO: World Health Organizations
